# Individually moulded silicone dressing in full thickness skin grafts

**DOI:** 10.1186/s40463-022-00577-7

**Published:** 2022-09-14

**Authors:** Michael Götting, Rahel Zibell, Markus Jungehülsing

**Affiliations:** Department of Otolaryngology-Head and Neck Surgery, Abteilung für HNO, Kopf- und Halschirurgie, Klinikum Ernst von Bergmann, Charlottenstraße 72, Potsdam, 14467 Germany

**Keywords:** Full thickness skin graft, Silicone dressing, Venylpolysiloxane

## Abstract

**Background:**

Full thickness skin grafting is a common technique for reconstructing defects in the head and neck area. We propose the use of an addition-cured silicone as an individually moulded silicone dressing to keep the vulnerable skin graft in place, prevent shearing forces and create a moist environment.

**Method:**

The silicone dressing is applied directly on the graft, using a double cartridge system. The silicone is moulded to the skin graft and hardens quickly, integrating thread knots into its material and creating good adherence to the graft. Charts of 24 patients who had undergone reconstruction with full thickness skin graft from the neck after surgery for skin tumors in the head from November 2017 to October 2020, were reviewed retrospectively to quantify the degree of post-operative graft loss and durability of the dressing.

**Conclusion:**

Medical silicone based on venylpolysiloxane is a safe and durable dressing which makes postoperative dressing changes expendable.

*Trial registration* The study was approved by the institutional review board of the Brandenburg state medical association (S 4(bB)/2021).

**Graphical Abstract:**

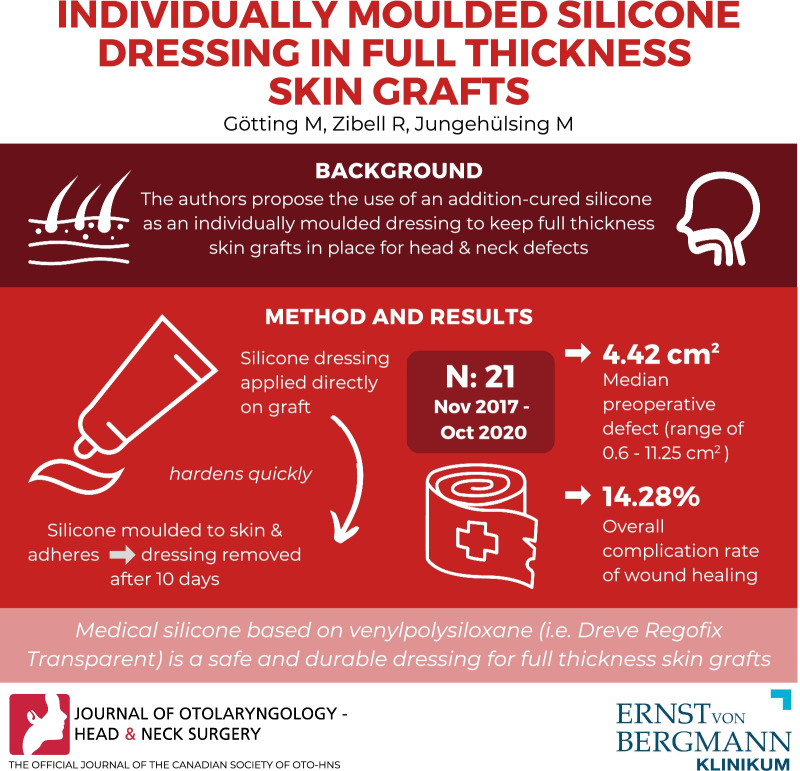

**Supplementary Information:**

The online version contains supplementary material available at 10.1186/s40463-022-00577-7.

## Introduction

Full thickness skin grafting (FTSG) is one of the most important techniques for reconstructing defects in head and neck skin. Grafts usually heal with good aesthetic and functional results, but require substantial nourishment [1].

In the first 24 h after placement the grafts are solely nourished by plasmatic imbibition. 48–72 h after grafting, revascularisation begins to develop when vascular anastomoses between the recipient bed and skin graft are being built. inosculation takes place five to seven days postoperative. If a graft’s metabolic demands are too high, or if the fragile connection of capillaries between the wound bed and graft is disrupted by shearing forces, the graft will die [2].

Due to the vulnerability to insufficient vascularization, it is important that the FTSG needs to be immobile during the time period of inosculation. This can be supported by a dressing that ideally keeps the skin graft in contact with the wound bed, increases the surface area contact, and helps prevent shearing [3].

In addition to mechanical protection, semi-occlusive and occlusive dressings prevent desiccation of the skin graft by creating a moist environment, which improves wound healing and prevents sticking of the sore edge.

Complex anatomical structures of the head and neck area, such as ears or nose, often make it difficult to place an occlusive dressing that allows monitoring of the graft and lasts a week or more, and therefore frequent dressing changes are necessary.

To improve this situation, we propose the use of an addition-cured, transparent and individually moulded silicone based on venylpolysiloxane (Dreve Regofix transparent), as a dressing applied directly on the graft. The silicone is a well-established impression material used in dentistry, and is widely available [4, 5].

## Material and methods

A retrospective medical chart review was undertaken to identify patients who had undergone FTSG in the head after surgery for skin tumors and received a semi-occlusive silicone dressing, at two institutions, from November 2017 to October 2020.

The study was approved by the institutional review board of the Brandenburg state medical association (S 4(bB)/2021).

Charts of twenty four patients were reviewed. To quantify the degree of post-operative graft loss, partial loss was defined as less than 50% of the graft surface being necrotic. Total graft loss was defined as loss of more than 50% of the surface.

## Surgical technique

**Fig. 1:**
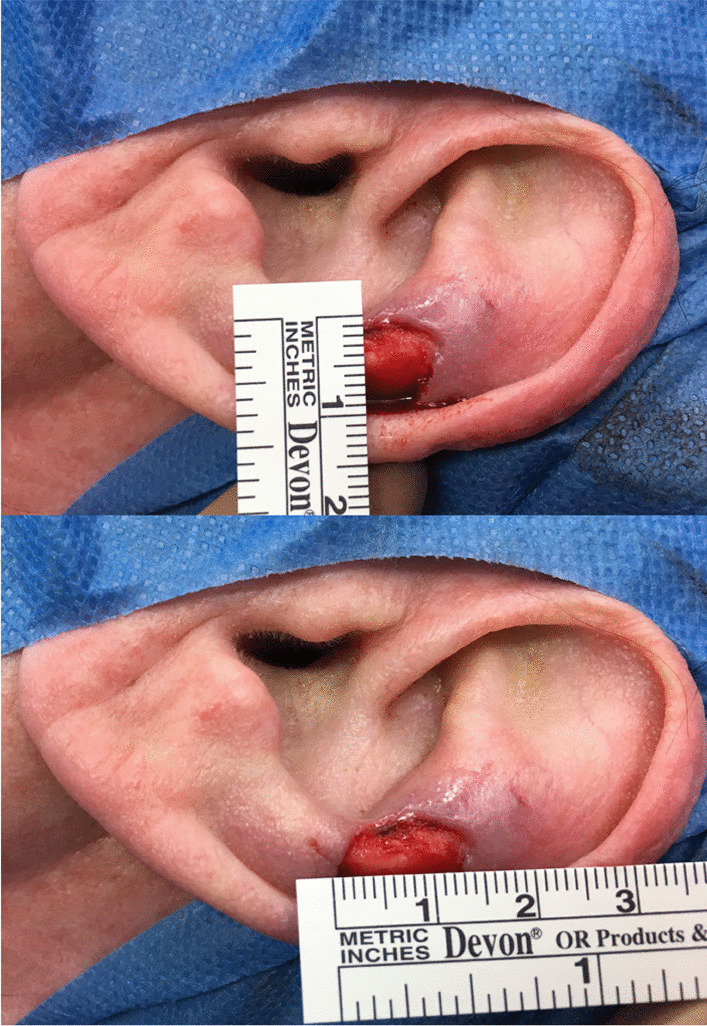
Preoperative defect size of the anthelix left (above and below)

The patient is informed about the off label use of a medical silicone. The procedure is performed under local anaesthesia. To cover a post-operative defect after histopathological R0-resection of a skin tumor in the head and neck region, a plastic reconstruction with FTSG is performed (Fig. 1).

**Fig. 2:**
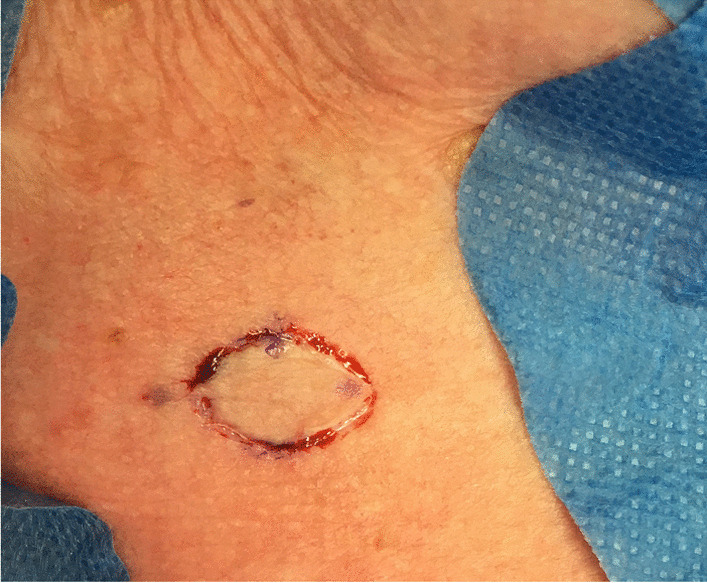
The full thickness skin graft ist harvested from the neck

FTSG are harvested from the neck in a shape fitting the defect, and the donor site wound is closed primarily with sub-dermal vicryl 4.0. sutures, topical skin adhesive (LiquiBand Optima) and steristrips (Fig. 2).

**Fig. 3:**
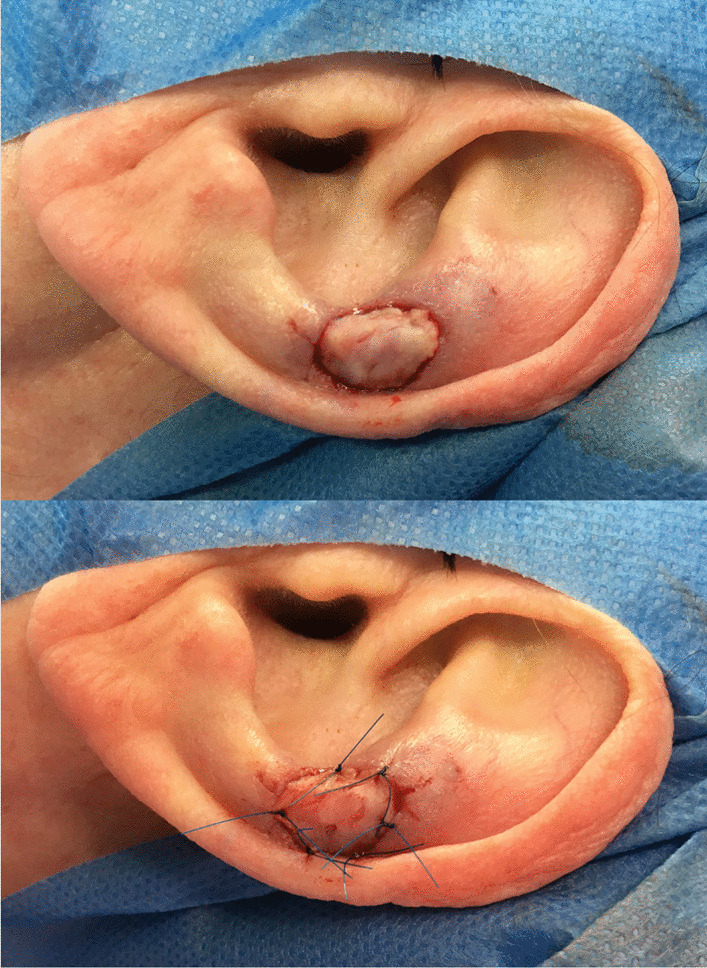
the prepared graft is fitted into the defect (picture above) and sutured tension free into place with a chromic 4.0 suture (picture below)

Sub-dermal fat is then removed from the graft, which is pierced with a no. 11 scalpel. After meticulous hemostasis to prevent hematoma of the recipient site, the prepared graft is sutured tension-free in place with a 4.0 chromic suture (Fig. 3).

**Fig. 4:**
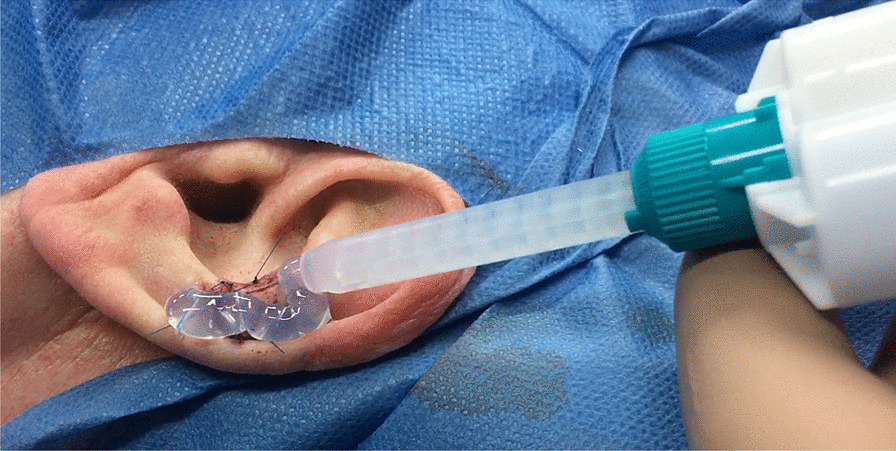
The graft is covered with addition cured silicon as a dressing

The graft is covered with a dressing of transparent addition-cured silicone (based on venylpolysiloxane) applied directly on the graft, using a double cartridge system with an injector and mixing tips for the right mix of components (Fig. 4).

**Fig. 5:**
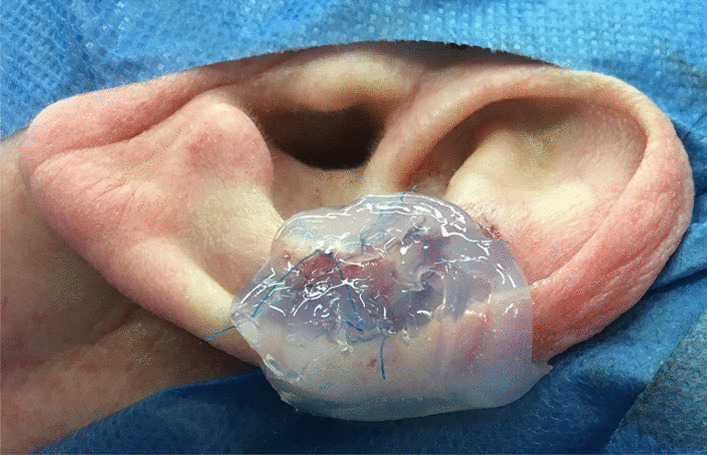
The moulded silicone is hardend and integrates the threads into its material

Application of the material takes only a few seconds, as the silicone is moulded to the skin graft and hardens quickly, integrating thread knots into its material and creating good adherence of the wound dressing. (Fig. 5).

**Fig. 6:**
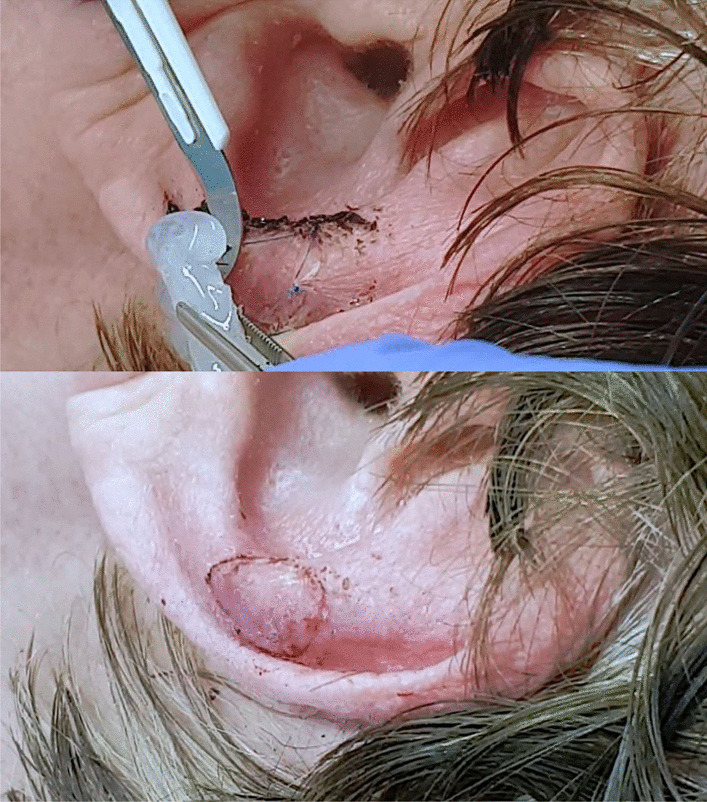
Removal of the dressing after ten days by cutting the threads at one side and lifting the dressing (picture above). After the complete removal of the dressing, the wound is cleaned (picture below)

After ten days, thread pull and removal of the dressing is a combined process, firstly by cutting the threads at one side and then lifting the dressing slowly by cutting all threads one by one. After complete detachment of the dressing, remaining threads are removed carefully and the wound is cleaned (Fig. 6).

## Results

Twenty four patients were treated with individually moulded silicone dressings after reconstruction of a defect in the head and neck region, with an FTSG from the neck, in the period from November 2017 to October 2020.

Three patients had incomplete charts and were excluded from the study, which analysed the medical records of 21 patients.

Median age of patients was 80.5, ranging from 58 to 95 years old. Males comprised 76.19% (*n* = 16) of patients and females 23.8% (*n* = 5).

Median preoperative defect size was 4.42 cm^2^, ranging from 0.6 to 11.25 cm^2^. Mean number of days until removal of the dressing as a proxy for the durability was 10.81, with a range from 6 to 18 days.

After removal of the dressing, partial graft loss occurred in two (9.52%) cases and a complete graft loss occurred in one (4.76%) case, leading to an overall complication rate of wound healing of 14.28%.

When analyzing the three patients with complications, the patient with complete graft loss had a preoperative size defect of 5.29 cm^2^ and the shortest duration of applied silicone dressing at 6 days. One of the patients with partial graft loss had a duration of 7 days with a defect size of 2.55 cm^2^. Whereas the other patient had an application duration of 11 days with a defect size of 6.6 cm^2^. Hence the median preoperative defect size within the patient group with complications didn’t differ significantly from the overall preoperative defect size among all analyzed cases (4.81 cm^2^ vs. 4.42 cm^2^).

Donor site for FTSG in all patients was the neck. In 61.9% of cases (*n* = 13) the recipient site was the ear (immobile but anatomically complex) and in 38% (*n* = 8) the recipient site was the mobile region of the face (nose, nasolabial fold and cheek). Mean duration of the dressing related to the localisation was 9.84 days in the ear and 12.22 days in the mobile region of the face which was statistically significant (*p* < 0.05). Preoperative defect size was 5.71 cm^2^ in the mobile region of the face and 3.52 cm^2^ in the ear.

There were no allergic reactions or intolerances to the dressing documented.

## Discussion

In the complex three dimensional surfaces of head and face anatomy, it is often challenging to place a semi occlusive dressing which is durable and doesn’t need frequent dressing changes. Moulded, addition-cured silicone serves as a semi occlusive dressing, durable on average for more than eleven days without a dressing change. It achieves the right amount of pressure and immobilisation for the graft to heal and allows direct visualisation of it.

In addition to the benefits for wound healing, the absence of dressing changes leads to a reduction of visits to the outpatient clinic, with resulting medico-economic savings and less logistical effort for the mostly elderly patients (mean age of 80.5 years in our study population) and physicians.

By analysing the patients with graft loss, there was no relation between the preoperative defect size and graft loss. In particular the patient with the largest FTSG had no problems with wound healing.

Up to now, silicone as a dressing is mostly used as a gel sheet for preventing and treating hypertrophic and keloid scars, and has proved useful and safe, although the exact mechanism is not completely understood yet [5, 6].

In our technique, the silicone is used as a transparent semi occlusive dressing that is moulded on complex wounds and leads to a moist chamber with increased temperature and improved hydration. Furthermore, it holds the FTSG in place and shearing forces are reduced which is especially important in FTSG.

Limitations in the method are possible complications from the material, such as intolerance or rashes, which were not seen in our study, possibly due to the small number of patients. Therefore, rare complications didn’t show up. In case of wound infection, bleeding or necrosis which makes removal of the dressing necessary, thread pull also has to be done, since the dressing holds on to the threads.

In comparison to traditional dressings with a bolster, such as petroleum or paraffin impregnated gauze with an overlying pressure dressing to secure the graft in place, it is not possible to monitor the graft without a dressing change and the application is rather complicated and time consuming. The application of the addition-cured silicone takes only a few seconds and the cartridge can be used several times with a new mixing tip for each patient.

The costs at our institution for a 50 ml cartridge are 8.01 € and 0.39 € for a mixing tip. The mixing tips are available in different sizes; the tip we use contains 1,6 ml and has to be discarded after use on one patient. The mean defect size in our collective is 4.42 cm^2^. Arithmetically 1.25 ml silicone cover 5 cm^2^ wound surface with a thickness of 2.5 mm, so that a 50 ml cartridge can be used 17.54 times.

This results in average cost of 0.85 € per application including the mixing tip for a wound surface of 5 cm^2^. In relation, bigger silicone dressings are more cost effective because of the high proportion of the mixing tip in the total cost.

In comparison, a traditional bolster dressing with Paraffin impregnated gauze (Lomatuell, 5 cm^2^) and overlying compress would cost 0.74 € at our institution. In bigger defects, an additional chromic suture can be necessary for the bolster dressing to hold effectively. This would exceed the costs of the moulded silicone dressing as chromic suture material is expensive (e.g. Prolene 4.0, 75 cm, 2.48 €).

## Conclusion

The use of addition-cured silicone as an individually moulded dressing is a safe technique to achieve a very durable, three dimensional dressing, with no need for regular postoperative dressing changes (Additional file 1).

Especially in bigger defects, silicone can be cheaper than traditional bolster dressings with potential cost savings for publicly funded health systems.

## Supplementary Information

**Additional file 1:** Supplemental video.

## Data Availability

The datasets used and analysed during the current study are available from the corresponding author on reasonable request.

## Abbreviation

FTSG: Full thickness skin grafting
